# Draft Genome Sequence of the Type Strain *Sphingopyxis bauzanensis* DSM 22271

**DOI:** 10.1128/genomeA.01014-17

**Published:** 2017-09-14

**Authors:** Michal A. Kaminski, Ewa M. Furmanczyk, Andrzej Dziembowski, Adam Sobczak, Leszek Lipinski

**Affiliations:** a Institute of Biochemistry and Biophysics, Polish Academy of Sciences, Warsaw, Poland; b Institute of Genetics and Biotechnology, Faculty of Biology, University of Warsaw, Warsaw, Poland

## Abstract

We present here the draft genome sequence of *Sphingopyxis bauzanensis* DSM 22271. The assembly contains 4,258,005 bp in 28 scaffolds and has a GC content of 63.3%. A series of specific genes involved in the catabolism or transport of aromatic compounds was identified.

## GENOME ANNOUNCEMENT

The members of the genus *Sphingopyxis* (family *Sphingomonadaceae*) have been isolated from chemically contaminated environments, mainly oil- and petrol-polluted soil and water (1, 2). Microorganisms from the family *Sphingomonadaceae* have the ability to use polycyclic aromatic hydrocarbons as a sole carbon source (3). Here, we present the draft genome sequence of *Sphingopyxis bauzanensis* type strain DSM 22271 (=BZ30, =CGMCC 1.8959, =CIP 110136), isolated from hydrocarbon-contaminated soil (4); this is the only representative genome of this species. Because it was collected from a contaminated environment, we were interested in genes encoding proteins responsible for hydrocarbon degradation.

Genomic DNA was isolated as previously described (5). Illumina paired-end (with an average insert size of 450 bp) and Nextera mate pair libraries (with an average insert size of 8 kb) were prepared according to the manufacturer’s protocols (a KAPA HTP DNA library preparation kit for Illumina sequencing and a Nextera mate pair sample prep kit, respectively). Whole-genome sequencing of *S. bauzanensis* strain DSM 22271^T^ was performed using the Illumina MiSeq platform (2 × 300 bp) and resulted in 487,322 paired reads for the paired-end library and 2,039,112 paired reads for the mate pair library. Reads from the paired-end library were processed as follows: adapters were removed using the Cutadapt script (6), and then the reads were filtered by length (>50 bp) and quality (*Q* value >30) (7). The mate pair reads were processed with NxTrim (8). Assembly was done using SPAdes version 3.9.1 (9). Contigs longer than 1 kb were deposited in GenBank and annotated using NCBI PGAP (10). The assembly consists of 28 scaffolds containing 4,258,005 bp with a GC content of 63.3%. The DSM 22271^T^ genome consists of 4,136 predicted genes, of which, 3,932 are protein-coding genes. The DSM 22271^T^ genome has 52 RNA genes, 46 tRNAs, 3 rRNAs, and 3 noncoding RNAs (ncRNAs), and 204 pseudogenes.

Twenty-six dioxygenases were predicted in the analyzed genome sequence, of which 13 were encoded on a single scaffold, number 6. This scaffold contains 68 open reading frames encoding proteins thought to be associated with the catabolism or active transport of aromatic compounds. Scaffold 6 is flanked with integrase-encoding genes, suggesting that it is a part of a catabolic transposon. Deeper analysis of proteins encoded on scaffold 6 showed their high similarity to proteins from a catabolic module described already on plasmid pNL1 from *Novosphingobium aromaticivorans* F199 (formerly *Sphingomonas aromaticivorans* F199) (11). The genes associated with biphenyl, xylene, and naphthalene degradation identified in *S. bauzanensis* strain DSM 22271^T^ were situated in a similar orientation to the pNL1 plasmid instead of in one major rearrangement. The genes *bphD*, *bphE*, and *bphF*, together with coenzyme A-transferase, were localized upstream of the *bphB* gene, separating the *bphB* sequence from *xylA*. Such gene rearrangements result in a concentration of the catabolic enzymes in the genome compared to pNL1.

Preliminary studies of the *S. bauzanensis* DSM 22271^T^ genome sequence suggest that this strain is well adapted for degradation of high-molecular-weight polycyclic aromatic hydrocarbons and has potential in the bioremediation of polluted environments.

### Accession number(s).

This whole-genome shotgun project has been deposited at DDBJ/ENA/GenBank under the accession no. NISK00000000. The version described in this paper is the first version NISK01000000.
